# An Intuitionistic Extension of the Simple WISP Method

**DOI:** 10.3390/e24020218

**Published:** 2022-01-29

**Authors:** Edmundas Kazimieras Zavadskas, Dragisa Stanujkic, Zenonas Turskis, Darjan Karabasevic

**Affiliations:** 1 Institute of Sustainable Construction, Vilnius Gediminas Technical University, Sauletekio al. 11, LT-10223 Vilnius, Lithuania; zenonas.turskis@vilniustech.lt; 2 Technical Faculty in Bor, University of Belgrade, 11000 Belgrade, Serbia; dstanujkic@tfbor.bg.ac.rs; 3 Faculty of Applied Management, Economics and Finance, University Business Academy in Novi Sad, 11000 Belgrade, Serbia; darjan.karabasevic@mef.edu.rs

**Keywords:** intuitionistic fuzzy set, simple WISP, singleton intuitionistic fuzzy number, MCDM

## Abstract

In this article, we present a new extension of the Integrated Simple Weighted Sum-Product (WISP) method, adapted for intuitionistic numbers. The extension takes advantage of intuitionistic fuzzy sets for solving complex decision-making problems. The example of contractor selection demonstrates the use of the proposed extension.

## 1. Introduction

Many decision-making problems are related to inaccuracies, unreliability, or predictions. Therefore, the significant development and use of multiple criteria decision making (MCDM) occurred after Zadeh [1] proposed the fuzzy set theory. Based on the fuzzy set theory, Bellman and Zadeh [2] proposed decision making in a fuzzy environment and thus enabled the use of MCDM for solving more complex decision-making problems. Indeed, the use of MCDM methods for solving decision-making problems in a fuzzy environment also required their adaptation to fuzzy sets.

The possibilities of fuzzy sets to apply crisp numbers influenced newly proposed extensions of the fuzzy set theory, such as interval-valued fuzzy (IVF) sets [3], intuitionistic fuzzy (IF) sets [4], neutrosophic set theory [5], and others. Based on the IVF and IF sets, Atanassov and Gargov [6] introduced interval-valued intuitionistic fuzzy (IVIF) sets. In the fuzzy set theory, Zadeh [1] introduced the membership function $\mu_{A}\left(x\right)$, which represents the belonging to the set, $\mu_{A}\left(x\right)\in \left[0,\;1\right].$ In IF set theory, Atanassov [4] extended the fuzzy set theory by introducing the non-membership function $\mu_{A}\left(x\right)$, $\nu_{A}\left(x\right)\in \left[0,\;1\right]$, with the following restriction $0\;\leq \mu_{A}\left(x\right)+v_{A}\left(x\right)\leq 1.$ The introduction of the non-membership function enabled the IF set theory to solve some decision-making problems that could not be easily solved by applying the FS theory.

Decision makers introduced many MCDM methods to solve complicated MCDM problems over time, such as ELECTRE [7], AHP [8], TOPSIS [9], COPRAS [10], VIKOR [11], MULTIMOORA [12,13], ARAS [14], WASPAS [15], and others. In addition to well-known MCDM methods, there are also newly proposed ones such as the EDAS [16], CODAS [17], CoCoSo [18], and MULTIMOOSRAL methods [19]. A comprehensive overview of the newly proposed MCDM methods, as well as their applications, can be found in Mardani et al. [20,21], Hafezalkotob et al. [22], Chandrawati et al. [23], and Liu and Xu [24].

IF sets had success in many problems, such as selecting knowledge management systems [25], assessing and ranking the risk of failure modes [26], choosing the right supplier [27], monitoring and continuous improving of an end-of-life vehicle management system [28], analyzing failure mode effects [29], and assessing solid waste management techniques [30]. Decision makers, to solve a much more comprehensive range of problems, proposed many extensions for almost all MCDM methods, such as TOPSIS [31,32], VIKOR [33], MULTIMOORA [34], and ARAS [35].

Decision makers have used the IVIF sets to assess reservoir flood control management [36], select the proper facility location [37], choose proper sustainable material [38], evaluate public transportation options [39], prioritize risks [40], rank choices of sustainable organizational development of companies [41], evaluate malicious code threats [42], and prioritize government roles in a merger and acquisition process [43]. Moreover, decision makers have used the IVIF sets to determine criteria weights [44,45,46]. Roszkowska et al. [47] also adopted the intuitionistic fuzzy TOPSIS for assessing social and economic phenomena. Similar to IFS, appropriate IVIF extensions are available for many MCDM methods, such as the COPRAS [48], WASPAS [36,49], ELECTRE [50], CODAS [37,38], TOPSIS [51,52], VIKOR [51], and CoCoSo [52] methods.

Stanujkic et al. [53] proposed the Integrated Simple Weighted Sum-Product (WISP) method. So far, there is no extension proposed for this method that allows its usage with IF sets, i.e., IF numbers.

Therefore, in this article, we suggest an extension of the WISP method, enabling IF numbers. The rest of the article is structured as follows. Section 2 explains the basic elements of IF sets. Section 3 presents the WISP method. Section 4 introduces an intuitionistic extension of the WISP method and proposes an IF-WISP method. Section 5 considers an example of contractor selection to illustrate the usage of the proposed extension. Section 6 compares the results obtained using the proposed approach and similar extensions of MCDM methods. The final section presents conclusions.

## 2. Preliminaries

This section presents some basic elements of IF sets.

### 2.1. The Basic Elements of Intuitionistic Fuzzy Sets

**Definition** **1.** *Let X be the universe of discourse. The IF set I in X is as follows [4]:*

(1) $$I=\left\{\;x<\mu_{I}\left(x\right),\nu_{I}\left(x\right),\;>|x\in \;Χ\right\},$$

*where* $\mu_{I}\left(x\right)$*denotes the extent of the membershipand* $\nu_{I}\left(x\right)$*denotes the extent of the non-membership of the element x to the set I,* $\mu_{I}\left(x\right),\;\nu_{I}\left(x\right)\;X\to \left[0,\;1\right]$*, and* $0\leq \mu_{I}\left(x\right)+\nu_{I}\left(x\right)\;\leq 1$.

Membership and non-membership functions can have different shapes such as trapezoidal, triangular, Gaussian, or the less commonly used singleton.

**Figure 1:**
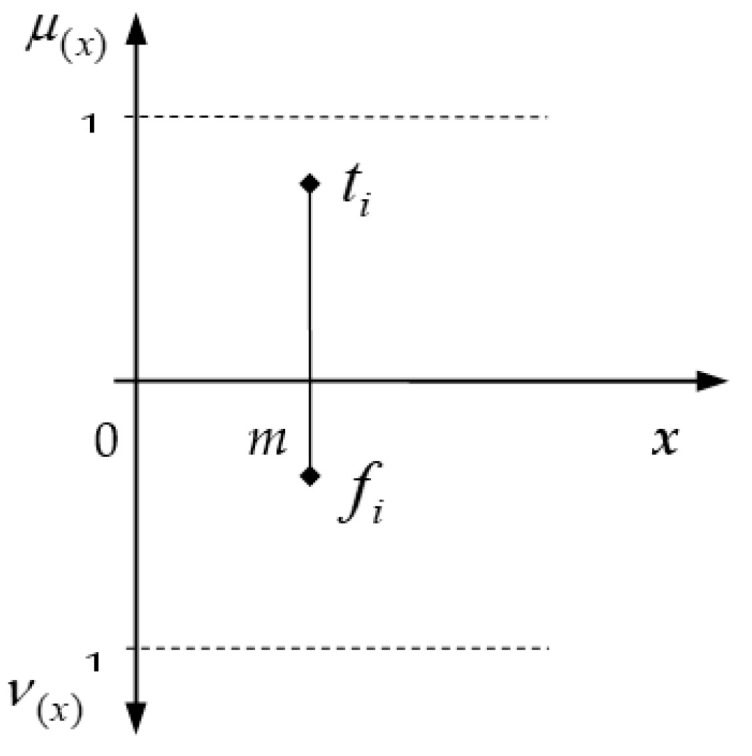
An SIF number.

**Definition** **2.** *A singleton intuitionistic fuzzy (SIF) number* $i=<t_{i},f_{i}>$ *, shown in Figure 1, is as follows:*

(2) $$\mu_{I\left(x\right)}=\{\begin{array}{cc} t_{i} & x=m\\ 0 & otherwise \end{array},$$

(3) $$\nu_{I\left(x\right)}=\{\begin{array}{cc} f_{i} & x=m\\ 0 & otherwise \end{array},$$

*where* m∈ℜ.

**Definition** **3.** *Let* $i_{1}=<t_{1},f_{1}>\;$*and* $i_{2}=<t_{2},f_{2}>$*be two IF numbers and* λ>0*. The basic operations on IF numbers are as follows:*

(4) $$i_{1}+i_{2}=<t_{1}+t_{2}-t_{1}t_{2},\;\;f_{1}f_{2}>,$$

(5) $$i_{1}\cdot i_{2}=<t_{1}t_{2},\;\;f_{1}+f_{2}-f_{1}f_{2},>,$$

(6) $$\lambda \cdot i_{1}=<1-{\left(1-t_{1}\right)}^{\lambda },\;\;f_{1}{}^{\lambda }>,$$

(7) $$i_{1}{}^{\lambda }=<t_{1}{}^{\lambda },\;1-{\left(1-f_{1}\right)}^{\lambda }>.$$

**Definition** **4.** *Let*$i=<t_{i},\;f_{i}>\;$*be an IF number. The score function s_(i)_ of i is as follows [54]:*

(8) $$s_{\left(i\right)}=t_{i}-f_{i},$$

*where* $s_{\left(i\right)}\in \left[1,-1\right]$.

**Definition** **5.** *Let*$I_{j}=<t_{j},\;\;f_{j}>$*be a collection of n SIF numbers. The intuitionistic fuzzy weighted arithmetic mean (IFWA) operator of I_j_ is as follows [55]:*

(9) $$IFWA_{\left(I_{j}\right)}=\sum_{j=1}^{n}I_{j}\;w_{j}=\left(1-\prod_{j=1}^{n}{\left(1-t_{j}\right)}^{w_{j}},\prod_{j=1}^{n}f_{j}{}^{w_{j}}\right).$$

*where w_j_ denotes the weight of element j of the collection A_j_,* $w_{j\;}\in \left[0,\;1\right],$*and* $\sum_{j=1}^{n}w_{j}\;$= 1.

**Definition** **6.** *Let* $I_{j}=<t_{j},\;\;f_{j}>$*be a collection of n SIF numbers. The intuitionistic fuzzy weighted geometric (IFWG) operator of I_j_ is as follows [55]:*

(10) $$IFWG_{\left(I_{j}\right)}=\prod_{j=1}^{n}A_{j}{}^{w_{j}}=\left(\prod_{j=1}^{n}t_{j}{}^{w_{j}},\;1-\prod_{j=1}^{n}{\left(1-f_{j}\right)}^{w_{j}}\right).$$

*where w_j_ denotes the weight of element j of the collection A_j_,* $w_{j\;}\in \left[0,\;1\right]$*and* $\sum_{j=1}^{n}w_{j}\;$= 1.

### 2.2. Deintuitionistification

At some stage in the MCDM process, it is necessary to transform the IF number into a crisp value. Decision makers can perform such a transformation using Equation (8). However, to perform a different analysis and consider different scenarios, a new approach for deintuitionistification, based on Equation (8), is proposed, as follows:

(11) $$s_{\left(i\right)}^{\lambda }=\lambda \;t_{i}-\left(1-\lambda \right)\;f_{i},$$

where *λ* represents coefficients, and λ ∈(0, 1).

## 3. The Simple Weighted Sum-Product Method

The procedure of the WISP method for a decision-making problem involving m alternatives that are evaluated based on n criteria is systemic procedure, the steps of which are as follows:

Step 1. Form a decision-making matrix and determine criteria weights.

Step 2. Construct a normalized decision-making matrix as follows:

(12) $$r_{ij}=\frac{x_{ij}}{\max_{i\;}x_{ij}},$$

where $r_{ij}$ denotes a dimensionless number representing normalized alternative *i* regarding criterion *j*.

Step 3. Calculate the values of four indicators, as follows:

(13) $$u_{i}^{sd}=\sum_{j\in \Omega_{\max}}r_{ij}w_{j}-\sum_{j\in \Omega_{\min}}r_{ij}w_{j},$$

(14) $$u_{i}^{pd}=\prod_{j\in \Omega_{\max}}r_{ij}w_{j}-\prod_{j\in \Omega_{\min}}r_{ij}w_{j},$$

(15) $$u_{i}^{sr}=\frac{\sum_{j\in \Omega_{\max}}r_{ij}w_{j}}{\sum_{j\in \Omega_{\min}}r_{ij}w_{j}},\;\mathrm{and}$$

(16) $$u_{i}^{pr}=\frac{\prod_{j\in \Omega_{\max}}r_{ij}w_{j}}{\prod_{j\in \Omega_{\min}}r_{ij}w_{j}},$$

where $u_{i}^{sd}$ and $u_{i}^{pd}\;$denote differences between the weighted sum and weighted product of normalized ratings of alternative i, respectively, and $\Omega_{\max}$ and $\Omega_{\min}$ denote sets of maximization and minimization criteria, respectively. Similar to the previous one, $u_{i}^{\mathrm{sr}}$ and $u_{i}^{\mathrm{pr}}$ denote ratios between the weighted sum and weighted product of normalized ratings of alternative *i*, respectively.

Step 4. Recalculate values of four indicators, as follows:

(17) $${\bar{u}}_{i}^{sd}=\frac{1+u_{i}^{sd}}{1+\max_{i\;}u_{i}^{sd}},$$

(18) $${\bar{u}}_{i}^{pd}=\frac{1+u_{i}^{pd}}{1+\max_{i}\;u_{i}^{pd}},$$

(19) $${\bar{u}}_{i}^{sr}=\frac{1+u_{i}^{sr}}{1+\max_{i}\;u_{i}^{sr}},\;\mathrm{and}$$

(20) $${\bar{u}}_{i}^{pr}=\frac{1+u_{i}^{pr}}{1+\max_{i}\;u_{i}^{pr}},$$

where ${\bar{u}}_{i}^{sd}$, ${\bar{u}}_{i}^{pd},\;{\bar{u}}_{i}^{sr}$, and ${\bar{u}}_{i}^{pr}$ denote recalculated values of $u_{i}^{sd}$, $u_{i}^{pd}$, $u_{i}^{sr}$, and $u_{i}^{pr}$.

Step 5. Determine the overall utility $u_{i}\;$of the considered alternative as follows:

(21) $$u_{i}=\frac{1}{4}\left({\bar{u}}_{i}^{sd}+{\bar{u}}_{i}^{pd}+{\bar{u}}_{i}^{sr}+{\bar{u}}_{i}^{pr}\right).$$

Step 6. Rank the alternatives and select the most suitable one. In this approach, the alternative with the highest value of *u_i_* is the most preferable.

The authors of the WISP method initially proposed using it to solve decision-making problems that contain both benefit- and cost-type criteria. However, the WISP method can also solve MCDM problems that contain only beneficial or only non-beneficial criteria, but in these cases, Equations (15) and (16) must be modified as follows:

(22) $$u_{i}^{sr}=\sum_{j\in \Omega_{\max}}r_{ij}w_{j},\;\mathrm{and}$$

(23) $$u_{i}^{pr}=\prod_{j\in \Omega_{\max}}r_{ij}w_{j},$$

when $\Omega_{min}=\emptyset$, that is:

(24) $$u_{i}^{sr}=\frac{1}{\sum_{j\in \Omega_{\min}}r_{ij}w_{j}},\;\mathrm{and},$$

(25) $$u_{i}^{pr}=\frac{1}{\prod_{j\in \Omega_{\min}}r_{ij}w_{j}},$$

when $\Omega_{max}=\emptyset$.

## 4. An Intuitionistic Extension of the WISP Method

To enable using the IFWG operator in the proposed IF extension of the WISP (IF-WISP) method, Equations (14) and (16), in the computational procedure of the standard WISP method, should be modified as follows:

(26) $$u_{i}^{pd}=\prod_{j\in \Omega_{\max}}r_{ij}{}^{w_{j}}-\prod_{j\in \Omega_{\min}}r_{ij}{}^{w_{j}},$$

(27) $$u_{i}^{pr}=\frac{\prod_{j\in \Omega_{\max}}r_{ij}{}^{w_{j}}}{\prod_{j\in \Omega_{\min}}r_{ij}{}^{w_{j}}},$$

After that, decision makers use the procedure of the IF-WISP method presented in the following steps:

Step 1. Construct an initial decision-making matrix. In this step, decision makers create an initial decision-making matrix that expresses the ratings of alternatives using IF numbers.

Step 2. Determine criteria weights. In this step, the criteria weights can be determined using any MCDM method primarily intended for determining the criteria weights, such as the AHP method [8], the SWARA method [56], or the Best-Worst method [57].

Step 3. Calculate the sum and product of the weighted intuitionistic ratings of each alternative for the maximization and minimization criteria, using Equations (9) and (10), as follows:

(28) $$S_{i}^{+}=\langle 1-\prod_{j\in \Omega_{\max}}{\left(1-t_{j}\right)}^{w_{j}},\;\;\prod_{j\in \Omega_{\max}}f_{j}{}^{w_{j}}\rangle ,\;$$

(29) $$S_{i}^{-}=\langle 1-\prod_{j\in \Omega_{\min}}{\left(1-t_{j}\right)}^{w_{j}},\;\;\;\;\prod_{j\in \Omega_{\min}}f_{j}{}^{w_{j}}\rangle ,$$

(30) $$P_{i}^{+}=\langle \prod_{j\in \Omega_{\max}}t_{j}{}^{w_{j}},\;1-\prod_{j\in \Omega_{\max}}{\left(1-f_{j}\right)}^{w_{j}}\rangle ,$$

(31) $$P_{i}^{-}=\langle \prod_{j\in \Omega_{\min}}t_{j}{}^{w_{j}},\;\;1-\prod_{j\in \Omega_{\min}}{\left(1-f_{j}\right)}^{w_{j}}\rangle ,$$

where $S_{i}^{\max}=<t_{i},f_{i}>\;$and $S_{i}^{\min}=<t_{i},f_{i}>\;$denote the sum of the weighted intuitionistic rating of alternative *i*, achieved based on maximization and minimization criteria, respectively, and $P_{i}^{\max}=<t_{i},f_{i}>\;$and $P_{i}^{\min}=<t_{i},f_{i}>\;$denote the product of the weighted intuitionistic ratings of alternative *i*, achieved based on maximization and minimization criteria, respectively.

Step 4. Deintuitionistification. The subtraction and division operations required for determining utility measures used in the WISP method are not primarily defined for IF set and IF numbers. Therefore, $S_{i}^{+},$ $S_{i}^{-},$ $P_{i}^{+},$ and ${\;P}_{i}^{-},$ should be transformed into crisp values using Equation (8) or Equation (11).

Step 5. Calculate the values of four indicators, $u_{i}^{\mathrm{sd}}$, $u_{i}^{\mathrm{pd}}$, $u_{i}^{\mathrm{sr}}$, and $u_{i}^{\mathrm{pr}}$, as follows:

(32) $$u_{i}^{sd}=S_{i}^{+}-S_{i}^{-},$$

(33) $$u_{i}^{pd}=P_{i}^{+}-P_{i}^{-},$$

(34) $$u_{i}^{sr}=\{\begin{array}{ll} \frac{S_{i}^{+}}{S_{i}^{-}} & when\;\Omega_{\max}\neq \emptyset \;\;\wedge \;\Omega_{\max}\neq \emptyset \\ S_{i}^{+} & when\;\Omega_{\min}=\emptyset \;\;\\ \frac{1}{S_{i}^{-}} & when\;\Omega_{\max}=\emptyset \end{array},\;\mathrm{and}$$

(35) $$u_{i}^{pr}=\{\begin{array}{ll} \frac{P_{i}^{+}}{P_{i}^{-}} & when{\;\Omega }_{\max}\neq \emptyset \wedge {\;\Omega }_{\max}\neq \emptyset \\ P_{i}^{+} & when{\;\Omega }_{\min}=\emptyset \\ \frac{1}{P_{i}^{-}} & when{\;\Omega }_{\max}=\emptyset \end{array}.$$

Step 6. Recalculate values of four indicators, as follows:

(36) $${\bar{u}}_{i}^{sd}=\frac{1+u_{i}^{sd}}{1+\max_{i\;}u_{i}^{sd}},$$

(37) $${\bar{u}}_{i}^{pd}=\frac{1+u_{i}^{pd}}{1+\max_{i}\;u_{i}^{pd}},$$

(38) $${\bar{u}}_{i}^{sr}=\frac{1+u_{i}^{sr}}{1+\max_{i}\;u_{i}^{sr}},\;\mathrm{and}$$

(39) $${\bar{u}}_{i}^{pr}=\frac{1+u_{i}^{pr}}{1+\max_{i}u_{i}^{pr}},$$

where ${\bar{u}}_{i}^{sd}$, ${\bar{u}}_{i}^{pd}$, ${\bar{u}}_{i}^{sr}$, and ${\bar{u}}_{i}^{pr}$ denote recalculated values of $u_{i}^{sd}$, $u_{i}^{pd}$, $u_{i}^{sr}$, and $u_{i}^{pr}$.

Step 5. Determine the overall utility $u_{i}\;$of each alternative as follows:

(40) $$u_{i}=\frac{1}{4}\left({\bar{u}}_{i}^{sd}+{\bar{u}}_{i}^{pd}+{\bar{u}}_{i}^{sr}+{\bar{u}}_{i}^{pr}\right).$$

Step 6. Rank the alternatives and select the most suitable one. Decision makers rank the alternatives in descending order and select the best with the highest *u_i_*.

## 5. A Numerical Example

In this section, we discuss the application of the proposed extension of the WISP method on the example of contractor selection.

Based on the example discussed in Turskis and Zavadskas [58], in this case, the evaluation of four contractors was performed based on the following criteria: production specifications (*C*_1_), financial position (*C*_2_), standards and relevant certificates (*C*_3_), commercial strength (*C*_4_), performance (*C*_5_), and delivery price (*C*_6_).

Table 1 shows an initial intuitionistic decision-making matrix.

Table 1 also shows the criteria weights and optimization directions.

Table 2 shows the weighted intuitionistic ratings of the maximization and minimization criteria for considered alternatives.

Table 3 shows crisp sums and products of the weighted intuitionistic ratings. In this case, decision makers used Equation (8) to deintuitionistificate, i.e., transform IF numbers into crisp values, but they can also use Equation (11).

Table 3 shows the values of four utility measures, $u_{i}^{\mathrm{sd}}$, $u_{i}^{\mathrm{pd}}$, $u_{i}^{\mathrm{sr}}$, and $u_{i}^{\mathrm{pr}}$, calculated using Equations (31)–(34).

Table 4 shows the recalculated values of four utility measures, ${\bar{u}}_{i}^{\mathrm{sd}}$, ${\bar{u}}_{i}^{\mathrm{pd}}$, ${\bar{u}}_{i}^{\mathrm{sr}}$, and ${\bar{u}}_{i}^{\mathrm{pr}}$, calculated using Equations (36)–(39), as well as the overall utility measures, calculated using Equation (40).

As can be concluded from Table 4, the alternative denoted as *A*_3_ is the most appropriate alternative.

**Figure 2:**
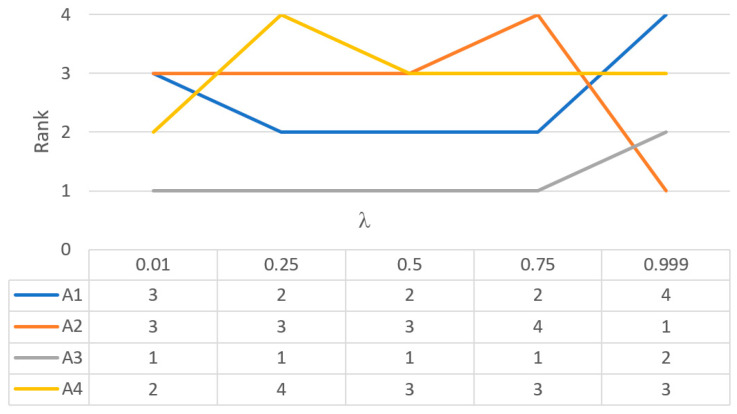
The ranking order of alternatives for different values of *λ*.

In addition to selecting the most appropriate alternative, the IF-WISP method allows analysis of the impact of membership and non-membership functions on the overall utility measures, using Equation (11). Table 5 and Figure 2 show the values of overall utility measures and ranks of alternatives for several selected values of the coefficient *λ*.

Based on the above, it is evident that the proposed IF-WISP extension decision makers can analyze different scenarios, thus making better use of the benefits that IF set theory provides for solving complex decision-making problems.

## 6. A Comparison of the Proposed Extension with Similar Extensions of Some MCDM Methods

In this section, we present tests of the proposed extension of the WISP method. We compared the obtained ranking results using the proposed extension with the results obtained using the neutrosophic WASPAS, CoCoSo, and SAW methods.

The authors chose the example discussed by Stanujkic et al. [59] to compare the ranking results. This example evaluated three alternatives based on four beneficial criteria: environment (*En*), content (*Co*), graphics (*Gr*), and authority (*Au*). Table 6 shows the ratings of the alternatives according to the evaluation criteria and the weights of the criteria.

Table 7 shows the ratings and ranking orders of alternatives obtained using intuitionistic extensions of the WASPAS, CoCoSo, SAW, and WISP methods.

As can be seen from Table 7, the ranking order of alternatives obtained using the proposed intuitionistic extension of the WISP method is the same as the ranking orders of alternatives obtained using the extensions mentioned above, which confirms the usability of the proposed extension.

## 7. Conclusions

Intuitionistic fuzzy sets provide an opportunity to solve more complex decision-making problems. The use of singleton intuitionistic fuzzy numbers is more straightforward than other intuitionistic fuzzy numbers (trapezoidal, triangular, or bell-shaped). However, they are still adequate to solve complex decision-making problems.

Therefore, we propose an extension of the WISP method adapted to use singleton intuitionistic fuzzy numbers (IF-WISP). The contractor selection problem demonstrates the usability of the newly proposed IF-WISP extension.

Finally, developing an interval-valued intuitionistic fuzzy extension of the WISP method can be stated as the future development direction. Furthermore, the development of similar fuzzy extensions, such as spherical, picture, and Pythagorean, can be mentioned as possible directions for further development of the WISP method.

## Figures and Tables

**Table 1:** An initial decision-making matrix.

	*C*_1_	*C*_2_	*C*_3_	*C*_4_	*C*_5_	*C*_6_
*w_j_*	0.210	0.137	0.137	0.131	0.175	0.210
Optimization	max	max	max	max	max	min
*A*_1_	<0.9, 0.0>	<0.7, 0.0>	<0.9, 0.0>	<1.0, 0.1>	<1.0, 0.0>	<1.0, 0.1>
*A*_2_	<0.9, 0.1>	<0.8, 0.1>	<1.0, 0.1>	<0.9, 0.0>	<0.8, 0.0>	<0.9, 0.1>
*A*_3_	<0.7, 0.0>	<1.0, 0.0>	<1.0, 0.0>	<1.0, 0.0>	<0.9, 1.0>	<0.9, 0.0>
*A*_4_	<0.8, 0.0>	<0.8, 0.1>	<0.9, 0.1>	<1.0, 0.0>	<1.0, 0.0>	<1.0, 0.2>

**Table 2:** Sums and products of weighted intuitionistic ratings of alternatives.

	$S_{i}^{+}$	$S_{i}^{-}$	$P_{i}^{+}$	$P_{i}^{-}$
*A*_1_	<0.08, 0.00>	<0.00, 0.62>	<0.92, 1.00>	<1.00, 0.38>
*A*_2_	<0.10, 0.00>	<0.02, 0.62>	<0.90, 1.00>	<0.98, 0.38>
*A*_3_	<0.09, 0.00>	<0.02, 0.00>	<0.91, 1.00>	<0.98, 1.00>
*A*_4_	<0.09, 0.00>	<0.00, 0.71>	<0.91, 1.00>	<1.00, 0.29>

**Table 3:** Crisp values of sums and products of weighted intuitionistic ratings.

	$S_{i}^{+}$	$S_{i}^{-}$	$P_{i}^{+}$	$P_{i}^{-}$	$u_{i}^{sd}$	$u_{i}^{pd}$	$u_{i}^{sr}$	$u_{i}^{pr}$
*A*_1_	0.08	−0.62	−0.08	0.62	0.70	−0.70	−0.13	−0.13
*A*_2_	0.10	−0.59	−0.10	0.59	0.69	−0.69	−0.17	−0.17
*A*_3_	0.09	0.02	−0.09	−0.02	0.07	−0.07	4.07	4.07
*A*_4_	0.09	−0.71	−0.09	0.71	0.80	−0.80	−0.12	−0.12

**Table 4:** The recalculated values of four utility measures, overall utility measures, and ranking order of alternatives.

	${\bar{u}}_{i}^{sd}$	${\bar{u}}_{i}^{pd}$	${\bar{u}}_{i}^{sr}$	${\bar{u}}_{i}^{pr}$	$u_{i}$	Rank
*A*_1_	0.94	0.32	0.17	0.17	0.402	2
*A*_2_	0.94	0.33	0.16	0.16	0.399	3
*A*_3_	0.59	1.00	1.00	1.00	0.898	1
*A*_4_	1.00	0.21	0.17	0.17	0.390	4

**Table 5:** The overall utility measures and ranking order of alternatives for different values of *λ*.

*λ*	0.01		0.25		0.5		0.75		0.999	
	$u_{i}$	Rank	$u_{i}$	Rank	$u_{i}$	Rank	$u_{i}$	Rank	$u_{i}$	Rank
*A*_1_	0.581	3	0.666	2	0.494	2	0.700	2	−5.021	4
*A*_2_	0.581	3	0.638	3	0.491	3	0.693	4	0.993	1
*A*_3_	0.755	1	0.697	1	0.934	1	0.961	1	0.967	2
*A*_4_	0.622	2	0.175	4	0.491	3	0.696	3	−4.596	3

**Table 6:** The ratings of alternatives and criteria weights.

	*C*_1_	*C*_2_	*C*_3_	*C*_4_
	*En*	*Co*	*Gr*	*Au*
*w_j_*	0.28	0.25	0.24	0.23
Optimization	max	max	max	max
*A*_1_	<0.742, 0.125>	<0.625, 0.375>	<0.590, 0.250>	<0.375, 0.250>
*A*_2_	<0.595, 0.327>	<0.750, 0.158>	<0.590, 0.125>	<0.500, 0.250>
*A*_3_	<0.717, 0.155>	<0.500, 0.125>	<0.586, 0.327>	<0.339, 0.176>

**Table 7:** The overall utility measures and ranking order of alternatives obtained using intuitionistic extensions of some MCDM methods.

	WASPAS	Rank	CoCoSo	Rank	SAW	Rank	WISP	Rank
*A*_1_	0.325	3	1.884	3	0.380	3	0.963	3
*A*_2_	0.300	1	2.164	1	0.419	1	1.000	1
*A*_3_	0.323	2	1.902	2	0.381	2	0.966	2
